# Response Comment on “Intestinal Helminths in Different Species of Rodents in North Khorasan Province, Northeast of Iran”

**Published:** 2019

**Authors:** Kourosh ARZAMANI, Mitra SALEHI, Iraj MOBEDI, Amir ADINEZADE, Hamid HASANPOUR, Mohammad ALAVINIA, Jamshid DARVISH, Mohammad Reza SHIRZADI, Zeinolabedin MOHAMMADI

**Affiliations:** 1. Vector-Borne Diseases Research Center, North Khorasan University of Medical Sciences, Bojnurd, Iran; 2. Department of Medical Parasitology, Faculty of Medicine, Gonabad University of Medical Sciences, Gonabad, Iran; 3. Department of Medical Parasitology and Mycology, School of Public Health, Tehran University of Medical Sciences, Tehran, Iran; 4. Department of Biology, Faculty of Sciences, Ferdowsi University of Mashhad, Mashhad, Iran; 5. Rodentology Research Department, Applied Animal Institute, Ferdowsi University of Mashhad, Mashhad, Iran; 6. Department of Zoonoses Control, Ministry of Health, Tehran, Iran

## Dear Editor-in-Chief

We would like to thank you for the opportunity to respond to the issues raised in Dr Borji’s letter. As mentioned in the article, the parasites were transferred to Parasite Laboratory, School of Public Health, Tehran University of Medical Sciences, Tehran, Iran for parasites identification. All specimens preserved in this laboratory.

Alimentary canals of rodents were removed and investigated for parasite infection. In some cases, (with obvious infection) the liver of the rodents was inspected for parasite infection.

In the last modification before publishing, format of all tables changed from right to left due to request of the journal office. Unfortunately, during these changes, a writing error occurred. Just titles of the tables were moved and the findings of this study in the table remained unchanged. We sent corrected table to the journal office previously.

As mentioned previously all rodents were stored at Ferdowsi University and Vector-Borne Diseases Research Center Museum of North Khorasan University of Medical Sciences, Iran and all parasites preserved in Laboratory of Parasitology of School of Public Health, Tehran University of Medical Sciences, Iran.

In the study, *Taenia taeniaeformis* was observed in the intestine of the rodents. This finding could be unattributed to the fact that the rodent may consume the cat remains while containing worms such as this. For example, if a dog feeds on a sheep’s liver infected with *Fasciola hepatica*, so it could be also reported as parasite found in a dog with similar explanation. So, the existence of *F. hepatica* in the dog’s intestinal is because the dog has feed from the sheep’s liver infected with this helminth.

**Best**

**Mitra SALEHI**

**Corresponding Author**

